# PD18 - Filaggrin loss-of-function variants are associated with clinical reactivity to foods

**DOI:** 10.1186/2045-7022-4-S1-P18

**Published:** 2014-02-28

**Authors:** Dorien Van Ginkel, GH Koppelman, BJ Kollen, S van der Heide, J Kukler, BMJ Flokstra-de Blok, AEJ Dubois

**Affiliations:** 1 Department of Pediatric Pulmonology and Pediatric Allergy, UMCG, GRIAC Research Institute, Groningen, The Netherlands; 2 Department of General Practice, UMCG, GRIAC Research Institute, Groningen, The Netherlands; 3 Department of Laboratory Medicine, UMCG, Groningen, The Netherlands

## Introduction

The mechanism that determines the difference between asymptomatic sensitisation and clinical reactivity to food is as yet unknown. The aim of this study was to determine the effect of loss-of-function variants in the *filaggrin* gene on clinical reactivity, sensitisation and severity of food allergy.

## Methods

Cases were defined as children with a positive DBPCFC to at least one food. Controls were defined as children with negative DBPCFCs to all foods tested. Specific IgE was measured by CAP-FEIA and a severity score was based on the symptoms during the DBPCFC. Four gene variants were genotyped: *R501XZ*, *S3247X*, *2282Del4* and *R2447X.*

## Results

We included 173 children and of these, 18 children were excluded due to Mendelian errors, low call rate, diagnostic indistinctness or non-western ethnicity.

The odds ratio for having loss-of-function variants of the *filaggrin* gene and being clinically reactive was 4.9, which corresponds to a relative risk of 1.5.

A history of eczema or specific IgE values did not change the beta coefficient of the effect of the *filaggrin* loss-of-function gene variants ≥10%, and both variables were therefore not considered to be confounders in this association.

A predictive model for clinical reactivity which included the presence of loss-of-function variants of the *filaggrin* gene had high specificity (98.1%) and positive predictive value (96.6%).

No associations were found between loss-of-function variants of the *filaggrin* gene and either sensitisation or the severity of food allergy.

## Conclusion and discussion

Of children suspected of being food allergic, those with loss-of-function variants of the *filaggrin* gene are 1.5 times more likely to be clinically reactive to a food than those carrying wild type alleles. This result is not confounded by eczema or specific IgE levels. These gene variants may help predict clinical reactivity in high risk children.

